# SIRT3 Protects Rotenone-induced Injury in SH-SY5Y Cells by Promoting Autophagy through the LKB1-AMPK-mTOR Pathway

**DOI:** 10.14336/AD.2017.0517

**Published:** 2018-04-01

**Authors:** Meng Zhang, Yong-Ning Deng, Jing-Yi Zhang, Jie Liu, Yan-Bo Li, Hua Su, Qiu-Min Qu

**Affiliations:** ^1^Department of Neurology, the First Affiliated Hospital of Xi’an Jiaotong University, Xi’an, China.; ^2^Center for Cerebrovascular Research, Department of Anesthesia and Perioperative Care, University of California, San Francisco, San Francisco, California, USA.

**Keywords:** Parkinson’s disease, SIRT3, Autophagy, α-synuclein, Oxidative stress, mitochondrial dysfunction

## Abstract

SIRT3 is a class III histone deacetylase that modulates energy metabolism, genomic stability and stress resistance. It has been implicated as a potential therapeutic target in a variety of neurodegenerative diseases, including Parkinson’s disease (PD). Our previous study demonstrates that SIRT3 had a neuroprotective effect on a rotenone-induced PD cell model, however, the exact mechanism is unknown. In this study, we investigated the underlying mechanism. We established a SIRT3 stable overexpression cell line using lentivirus infection in SH-SY5Y cells. Then, a PD cell model was established using rotenone. Our data demonstrate that overexpression of SIRT3 increased the level of the autophagy markers LC3 II and Beclin 1. After addition of the autophagy inhibitor 3-MA, the protective effect of SIRT3 diminished: the cell viability decreased, while the apoptosis rate increased; α-synuclein accumulation enhanced; ROS production increased; antioxidants levels, including SOD and GSH, decreased; and MMP collapsed. These results reveal that SIRT3 has neuroprotective effects on a PD cell model by up-regulating autophagy. Furthermore, SIRT3 overexpression also promoted LKB1 phosphorylation, followed by activation of AMPK and decreased phosphorylation of mTOR. These results suggest that the LKB1-AMPK-mTOR pathway has a role in induction of autophagy. Together, our findings indicate a novel mechanism by which SIRT3 protects a rotenone-induced PD cell model through the regulation of autophagy, which, in part, is mediated by activation of the LKB1-AMPK-mTOR pathway.

Parkinson’s disease (PD) is the second most common neurodegenerative disorder, affecting 1-3% of the population older than 50 years and more than 5 million people worldwide [1]. It is mainly characterized by a substantial loss of dopamine-containing neurons in the substantia nigra pars compacta. Previous studies have shown that accumularion of α-synuclein, oxidative stress and mitochondrial dysfunction are involved in the pathogenesis of PD[2]. These factors interact with and intensify each other and ultimately lead to neuronal death. Therefore, an ideal therapy should be able to address these problems simultaneously [3].

Autophagy, or cellular self-digestion, is a cellular pathway involved in protein and organelle degradation, with an astonishing number of connections to human disease and physiology. There are various types of autophagy, including macro- and microautophagy, as well as chaperone-mediated autophagy (CMA), and they differ in mechanisms and functions. More and more evidence has shown that autophagy plays a protective role in neurodegenerative diseases [4-6]. Evidence shows that α-synuclein could be degraded both by macroautophagy and CMA [7, 8]. In agreement with this pivotal role of autophagy in α-synuclein clearance, overexpression of Beclin 1 reduces α-synuclein aggregation and neuronal pathology [9]. In addition, autophagy plays important roles in preserving mitochondria function and oxidative stress control. Mitochondrial dynamics are crucial in preventing potential damage generated by ROS. Damaged mitochondrial degradation by autophagy is called mitophagy. Thus, mitophagy is of great importance in blocking the production of ROS [10]. Autophagy enhancement acts as an adaptive metabolic response to prevent increased levels of reactive oxygen species and cell death [11]. It was reported that autophagy-related gene knockout mice accumulate ROS and dysfunctional mitochondria with altered morphology [12]. Correspondingly, ROS-induced mitochondrial damage may also be an important upstream activator of mitophagy [13]. Taken together, disruption of autophagy may cause accumulation of α-synuclein aggregates in neurons[14], slow the degradation of damaged mitochondria and thus, increase the level of oxidative stress [15]. Conversely, up-regulation of autophagy may alleviate these pathologies that are linked to PD.

Sirtuins are conserved proteins with an NAD+-dependent deacetylase and mono-ADP-ribosyltransferase activity (class III histone deacetylases), including seven different proteins (SIRT1-SIRT7) in mammals. SIRT3 is a mitochondrial deacetylase that is enriched in highly metabolic tissues, such as the liver, heart, brain and brown adipose tissue. SIRT3 has been reported to regulate almost every aspect of the mitochondria [16]. Our previous study demonstrated that SIRT3 protects neuronal cells from rotenone-induced toxicity, reduces α-synuclein aggregation, and ameliorates oxidative stress [17]. However, further studies are necessary to determine the mechanism of these protective effects. SIRT3 also acts as an upstream or direct regulator in promoting autophagy [18-22]. SIRT3 has been shown to activate the LKB1-AMPK signaling pathway in a cardiac hypertrophy mouse model [23]. AMPK may act as an activator to promote autophagy via mTOR or through an interaction with ULK1 [24]. To our knowledge, no reports about SIRT3, PD and autophagy have been put forward so far.

Jang et al. established an in vitro PD model by applying rotenone on SH-SY5Y cells and found various neuroprotective substances with autophagic modulation properties, such as erythropoietin [25] and 1,25-Dyhydroxyvitamin D(3) [26]. In this study, the same in vitro PD model was used. We hypothesized that the neuroprotective effect of SIRT3 may be through the induction of autophagy via the LKB1-AMPK-mTOR signaling pathway.

## MATERIALS AND METHODS

### Materials

The rotenone, puromycin, 3-Methyladenine (3-MA), Dorsomorphin, 2’, 7’-dichlorodihydrofluorescein diacetate (DCFH-DA), 3-(4, 5-dimethylthiazol-2-yl)-2, 5-diphenyltetrazolium bromide (MTT) and retinoic acid (RA) were purchased from Sigma-Aldrich (St. Louis, MO, USA). The Bafilomycin A1 was obtained from Aladdin (Shanghai, China). The PE Annexin V Apoptosis Detection Kit was purchased from the Becton-Dickinson Company (Franklin lakes, NJ, USA). The GSH Assay Kit and SOD Assay Kit were provided by the Nan-Jing Jiancheng Biocompany (Nanjing, Jiangsu, China). The MMP Assay Kit with JC-1 was from Beyotime (Haimen, Jiangsu, China). The antibodies were purchased from the following companies: alpha-synuclein from the Becton-Dickinson Company (Franklin lakes, NJ, USA); LKB1, p-LKB1 (Ser428), AMPK, p-AMPK (Thr172), and Beclin 1 from Cell Signaling Technology (Danvers, MA, USA); LC3 from Sigma-Aldrich (St. Louis, MO, USA); mTOR (S2442) and p-mTOR (S2448) from Bioworld (St. Louis Park, MN, USA); β-actin from Santa Cruz Biotechnology (Santa Cruz, CA, USA); goat anti-mouse IgG secondary antibody and goat anti-rabbit IgG secondary antibody (both peroxidase conjugated) were purchased from Signalway Antibody (College Park. MD, USA). A goat anti-mouse IgG (Cy3-conjugated) was purchased from Zhuangzhi Biotechnology (Xi’an, China).

### Cell culture and drug treatment

The human neuroblastoma cell line, SH-SY5Y, obtained from American type culture collection (Manassas, VA, USA) was cultured in DMEM/F12 medium (Hyclone, Logan, UT), supplemented with heat-inactivated 10% fetal bovine serum (Biological Industries, Kibbutz Beit Haemek, Israel), in a humidified atmosphere containing 5% CO2 at 37°C.

**Figure 1.**
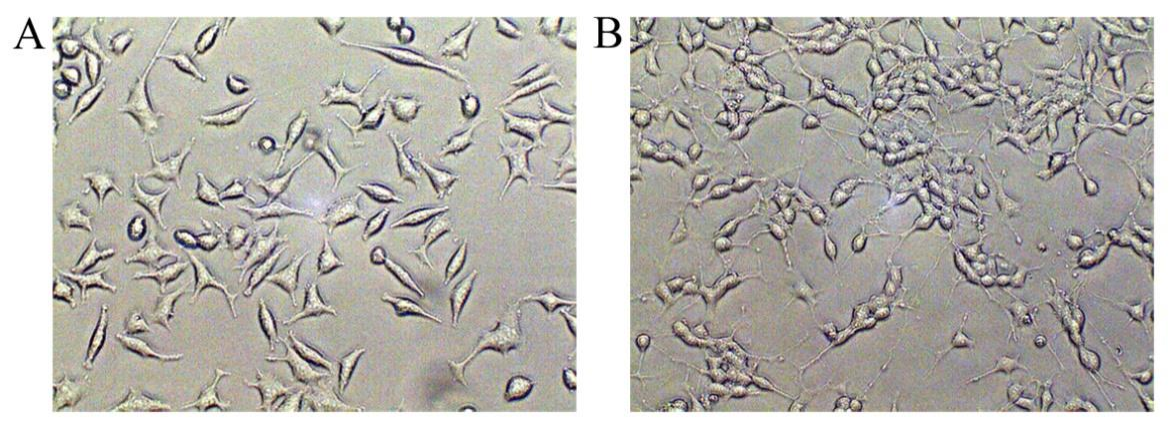
Differentiation of SH-SY5Y cells. (A) Undifferentiated SH-SY5Y cells cultured in 10% complete medium for 5 days. (B) Differentiated SH-SY5Y cells cultured in 1% cultural medium with RA (10 μM) for 5 days.

To induce differentiation, the SH-SY5Y cells were treated with with RA (10 μM) in 1% FBS medium in the dark for 5 days. The short processes in the undifferentiated state developed into long neurites in SH-SY5Y cells, reflecting a neuronal phenotype (Fig. 1).

The 3-MA was dissolved in sterile PBS with heating. The rotenone, Dorsomorphin and Bafilomycin A1 were dissolved in DMSO (final concentration of DMSO was 0.01%). The 3-MA (5mM) was added 1h prior to rotenone treatment. Rotenone (60μM) was used to incubate with cells for 24h to induce cell damage. Dorsomorphin (40μM) and Bafilomycin A1 (100nM) took 100min to take effect on the cells. To investigate whether SIRT3 could induce autophagy or not, 6 groups were established: WT-group, vehicle-group, SIRT3-group, WT+ Bafilomycin A1-group, vehicle+ Bafilomycin A1-group, and SIRT3+ Bafilomycin A1-group. To clarify whether autophagy is involved in the neuroprotective effect of SIRT3, the cells were divided into 9 groups: WT-group (SH-SY5Y cells without lentivirus infection), WT+Rot-group, WT+3-MA-group, Vehicle-group (lentivirus vector infection without targeting genes), Vehicle+Rot-group, Vehicle+3-MA-group, SIRT3-group (lentivirus infection with SIRT3 overexpression genes), SIRT3+Rot-group, and SIRT3+Rot+3-MA-group. To examine the LKB1-AMPK-mTOR pathway, 6 groups were generated: WT-group, vehicle-group, SIRT3-group, WT+ Dorsomorphin-group, vehicle+ Dorsomorphin-group, and SIRT3+ Dorsomorphin-group.

### Lentiviral infection of SH-SY5Y cells

To overexpress or silence SIRT3, LV-SIRT3 (6848-2) or LV-SIRT3i lentiviral vectors were produced, respectively, with Puromycin-resistance genes (Shanghai Genechem Co. Ltd.). For the overexpression of SIRT3, primers specific to the human SIRT3 gene were synthesized, and the sequences were as follows: (a) SIRT3-P1: CCAACTTTGTGCCAACCGGTCGCCACCATGGCGTTCTGGGGTTGG; (b) SIRT3-P2: AATGCCAACTCTG AGCTTTTTGTCTGGTCCATCAAGC. Four shRNAs with different target sequences are listed as follows: ACCTGCACAGTCTGCCAA (SIRT3-RNAi-1), GGCT CTACACGCAGAACAT (SIRT3-RNAi-2), CAACGT CACTCACTACTTT (SIRT3-RNAi-3), and GGGTGCT TCAAGTGTTGTT (SIRT3-RNAi-4). Both the SIRT3 overexpression and silencing genes were designed to carry puromycin-resistance genes and produced using the Lentivector Expression System (Shanghai Genechem Co. Ltd.). The SH-SY5Y cells were plated into 96-well culture plates (5000/well) and infected with lentivirus (MOI=10) on the day following when the cells reached 80% confluence. Stable singal clones were selected following 4-8 weeks of puromycin treatment (2μg/μL). SIRT3 expression in the stably infected clones was assessed using western blot analysis. One of the four shRNAs was selected by western blotting with the highest silencing efficiency[27].

### Cell viability assessment measured by the MTT assay

The SH-SY5Y cells were seeded into 96-well culture plates at a density of 8000/well in 0.2ml of culture medium. After being treated with different testing agents, the cells were incubated together with 5 mg/ml MTT for 4 h at 37°C. Then, the medium in each well was carefully aspirated, followed by addition of 150μl of DMSO in each well. The crystals were dissolved in DMSO and quantified by measuring the absorbance at 490nm using an automated micro-plate reader (BIO-TEK, VT, USA).

### Apoptosis analysis

Apoptosis of the cells was detected using the PE Annexin V Apoptosis Detection Kit (BD Pharmingen). After being treated with different testing agents, the cells were collected and washed twice with cold phosphate buffered saline (PBS), centrifuged at 1200 rpm for 5 minutes, and resuspended in 1X binding buffer at a concentration of 1×10^6^ cells/ml. One hundred microliters of the above solution (1×10^5^ cells) were transferred to a 10ml centrifuge tube and 5 μl PE Annexin V and 5 μl 7-AAD was added to the solution. The cells were gently vortexed and incubated for 15 minutes at room temperature in the dark, and then, 400μl of binding buffer was added to each tube. Apoptosis was measured by a FACScan flow cytometer (Becton Dickinson). The PE Annexin V -positive, 7-AAD-negative cells were scored as early apoptotic cells, while both positive for PE Annexin V- and 7-AAD-positive cells were considered to be late apoptotic cells.

### Detection of SOD, GSH

SOD and GSH were detected using the corresponding kits. After being treated with the indicated drugs, the cells were harvested, suspended in PBS and then fragmented with ultrasonic liquid processors (Sonics). The SOD and GSH in the suspension were analyzed according to the manufacturer’s instructions. For the detection of SOD and GSH, 450 nm and 405 nm were used, respectively, using an automated microplate reader (BioTek).

### Detection of ROS and MMP by Flow Cytometer

The ROS was detected using DCFH-DA, and MMP was with the assay kit. After various treatments of each group, the cells were harvested with trypsin, then suspended in PBS, and immediately stained with DCFH-DA or JC-1 according to the manufacturer’s instructions. Then, the samples were analyzed with a flow cytometer (FACScan flow cytometer, Becton Dickinson).

### Immunofluorescence staining

The SH-SY5Y cells were seeded on cover slips in a 24-well plate. After being treated with the indicated drugs, the cells on the cover slips were fixed in 4% paraformaldehyde for 30 min, washed with PBS, permeabilized with 0.1% Triton-X100, blocked in 5% BSA in PBS, and incubated with anti-alpha-synuclein (1:100) at 4°C overnight. Then, the cells were washed with PBS and incubated with Cy3-conjugated goat-anti-mouse IgG (1:100) at 37°C for 1 h, followed by staining with DAPI. The slides were subsequently mounted using glycerin and were observed under a confocal microscope (Olympus, Tokyo, Japan). The images were analyzed using ImageJ.

### Immunoblotting

The SH-SY5Y cells were washed twice with ice-cold PBS and then lysed in RIPA lysis buffer containing a protease inhibitor cocktail (Roche, Munich, Germany) and a phosphorylase inhibitor cocktail (Roche, Munich, Germany). After centrifugation at 4°C, the extracted proteins were quantified by the BCA kit (Beyotime, Jiangsu, China), and 100μg of protein from each group was loaded onto a 12% SDS-PAGE gel. After electrophoresis, the proteins were transferred onto PVDF membranes. Non-specific protein binding was blocked with 5% non-fat milk in PBS for 2 h at room temperature. The membranes were incubated with anti-LC3 (1:1,000), anti-Beclin 1 (1:800), anti-LKB 1 (1:600), anti-p-LKB1 (1:600), anti-AMPK (1:600), anti-p-AMPK (1:600), anti-mTOR (1:500), anti-p-mTOR (1:500),anti-α-synuclein (1:250) and anti-β-actin (1:1,0000) overnight at 4°C. After washing with PBST, the membranes were incubated with secondary antibodies conjugated with HRP at room temperature for 1 h. The signal was detected using an enhanced chemiluminescence detection kit (Millipore, USA). The immunoreactive bands of proteins were scanned with chemidoc XRS (Bio-Rad), and the digitized data were quantified as area and percentage of each band using ImageJ software.

### Electron microscopy

To confirm the autophagosome structures of autophagy, WT, Vehicle and SIRT3-overexpression SH-SY5Y cells were fixed with 2% glutaraldehyde for 2 h, washed with PBS and then post-fixed with 1% OsO_4_ for 1.5 h at 48°C. The samples were then washed and dehydrated with graded alcohol. After dehydration, the samples were infiltrated and embedded in 618 epoxy resin. Ultrathin sections were cut, stained with uranyl acetate and lead citrate and then examined under the transmission electron microscope H7650 (Hitachi, Tokyo, Japan).

### Statistical analysis

Significant differences among groups were determined with one-way analysis of variance (ANOVA) followed by a Tukey test using statistical software SPSS version 18.0. The data are presented as the mean ± SEM using Graphpad Prism (Graph Pad, San Diego, CA). The *P* values less than 0.05 were considered significant. All results were confirmed from a minimum of three independent experiments.

**Figure 2.**
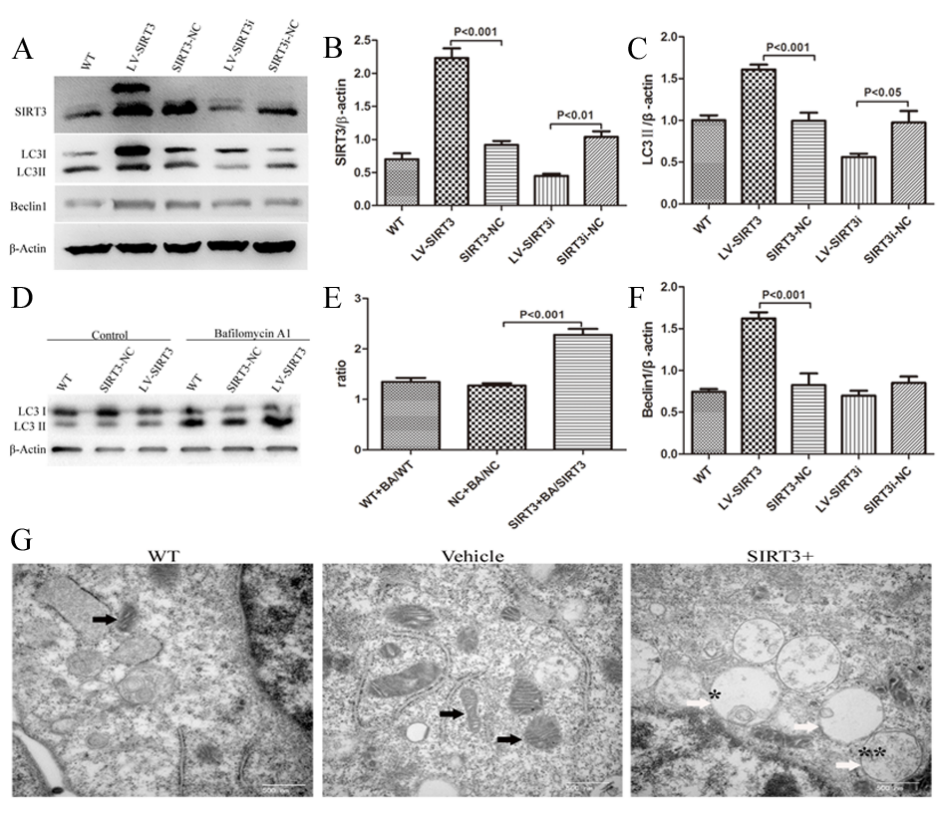
SIRT3 increases autophagy in human neuroblastoma SH-SY5Y cells. Lysates from cells untreated, or treated with SIRT3-NC or SIRT3i-NC (non-targeting lentivirus), LV-SIRT3 (Lentivirus with a SIRT3 overexpression gene) or LV-SIRT3i (Lentivirus with a SIRT3 silencing gene) were prepared and analyzed by western blotting. (A) SIRT3 overexpression and silencing using lentiviruses were identified by SIRT3 immunoblotting with an antibody against SIRT3. Autophagy induction by SIRT3 was determined by the LC3II and Beclin1 protein levels with an antibody against LC3B and Beclin 1. β-actin is used as a loading control. Mean ± SEM, n=3. Bar graphs show the quantification of the relative levels of SIRT3 (B), LC3II (C) and Beclin 1 (F). (D) SH-SY5Y cells with or without SIRT3 overexpression were treated with 100 nM Bafilomycin A1 for 100 min. Cell lysates were prepared and analyzed by western blotting. (E) Bar graphs show the quantification of LC3II level ratios between WT+BA and WT, Vehicle+BA and Vehicle, SIRT3+BA and SIRT3 groups. β-actin is used as a loading control. Mean ± SEM, n=3. (G) Ultrastructural SH-SY5Y cells in WT, Vehicle (NT-lentivirus) and LV-SIRT3 (SIRT3+) groups. Black arrows indicate mitochondria. White arrows show different stages of autophagic vascuoles: * represents an early autophagic vacuole (AVi); ** represents a degradative autophagic vacuole (AVd). An AVi can be identified by its contents (morphologically intact cytoplasm). The AVd contains partially degraded contents. Scale bars, 500 nm.

## RESULTS

### SIRT3 up-regulates autophagy in the SH-SY5Y cell line

Upon differentiation, SH-SY5Y cells possess more biochemical, ultrastructural, morphological and electrophysiological similarity to neurons, resulting in a more ideal PD cell model. Thus, the SH-SY5Y cells used in each experiment were differentiated cells. After performing lentiviral infection, we performed western blotting analysis to test the infection efficiency. SIRT3 expression increased significantly in the LV-SIRT3 group and decreased substantially in LV-SIRT3i cells (Fig. 2A, 2B). Immunoblotting for the most widely monitored autophagy-related protein, Atg8/LC3, indicated a significant increase of LC3II expression in the SIRT3 overexpression-group compared with the Vehicle-group and a remarkable decrease in the LV-SIRT3i group (Fig. 2A, 2C). To test whether SIRT3 increased LC3II expression by accelerating autophagy levels or by disrupting lysosomal degradation, we next delivered Bafilomycin A1 (100 nM, 100 min) to cells, and determined LC3II expression levels again. The LC3II levels in the WT, Vehicle and SIRT3 groups all increased dramatically after Bafilomycin A1 treatment, with the SIRT3+Bafilomycin A1-group displaying the greatest increase (Fig. 2D, 2E). Beclin 1 is vital in signaling the onset of autophagy. We found that Beclin 1 expression in the SIRT3 group increased significantly compared with the Vehicle group (Fig. 2A, 2F). The autophagosome, which is responsible for the sequestration of cytoplasm for degradation, is the morphological hallmark of autophagy. Thus, transmission electron microscopy was next used to examine the structure of the autophagosomes in SIRT3-group cells compared with the Vehicle controls (Fig. 2G). The SIRT3 overexpression group had more formation of autophagosomes, which are structured with double-membranes, than the Vehicle cells and WT cells. In conclusion, these data confirm the upregulation of autophagy by SIRT3 overexpression in the SH-SY5Y cell line.

**Figure 3.**
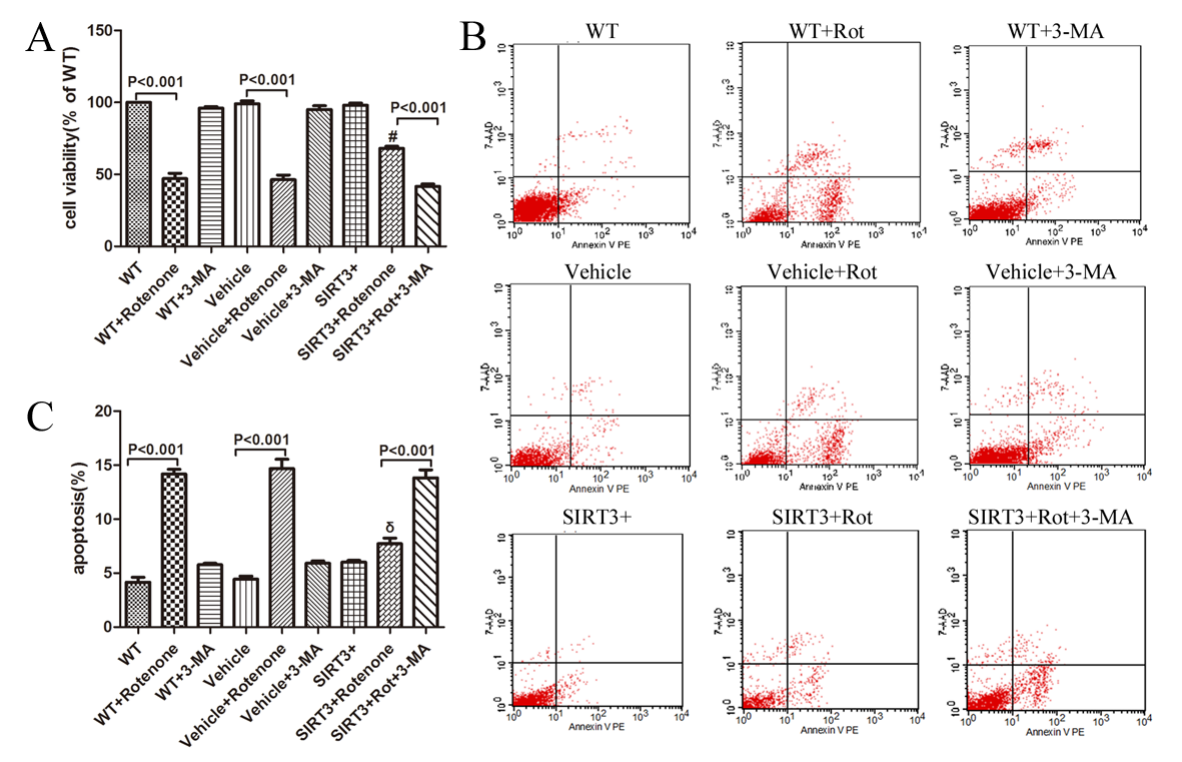
SIRT3 prevents rotenone-induced cell death in human neuroblastoma SH-SY5Y cells by upregulating autophagy. The SH-SY5Y cells with or without SIRT3 overexpression cultured in non-serum growth medium were treated with 60 μM rotenone for 24 h. Five millimolar 3-MA was added 1 h before rotenone. (A) Then, the treated cells were subjected to the MTT assay. Bar graph shows the quantification of cell viability determined by MTT assay in WT, WT+Rot, WT+3-MA, Vehicle, Vehicle+Rot, Vehicle+3-MA, SIRT3+, SIRT3+Rot, and SIRT3+Rot+3-MA groups. Mean ± SEM, n=3. #: *P*<0.001 vs. Vehicle+Rotenone. After treatment as described above, cells were harvested, stained with 7-AAD (Y-axis) and PE-Annexin V (X-axis). Scatter diagram (B) and bar graph (C) show the flow cytometric analysis of staining from WT, WT+Rot, WT+3-MA, Vehicle, Vehicle+Rot, Vehicle+3-MA, SIRT3+, SIRT3+Rot, and SIRT3+Rot+3-MA groups. The apoptotic rate= [AnnexinV-PE(+)7-AAD(-)cells + AnnexinV-PE(+)7-AAD(+)cells]/total cells×100%. Mean ± SEM, n=3. Rot=Rotenone. δ: *P*<0.001 vs. Vehicle+Rotenone.

### SIRT3 protects SH-SY5Y cells from rotenone toxicity through up-regulating autophagy

The addition of rotenone (60 μM, 24h) to the cell culture medium reduced the cell viability to approximately 50% in the WT+Rotenone-group and the Vehicle+Rotenone-group, while treatment with rotenone on SIRT3+ cells caused the cell viability to decrease approximately 30%, indicating that SIRT3 overexpression preserved cell viability (Fig. 3A). However, 3-MA could dramatically ameliorate the protective effect of SIRT3 on rotenone-induced cell damage (Fig. 3A). After the cell viability was measured by the MTT assay, we next quantified the apoptosis rate of each group, and obtained similar results, where SIRT3 protects SH-SY5Y cells from apoptosis after rotenone treatment, and the protective effect of SIRT3 was again abolished by 3-MA (Fig. 3B, 3C). These findings suggest the involvement of autophagy in the neuroprotectiive effects of SIRT3 on the PD cell model.

**Figure 4.**
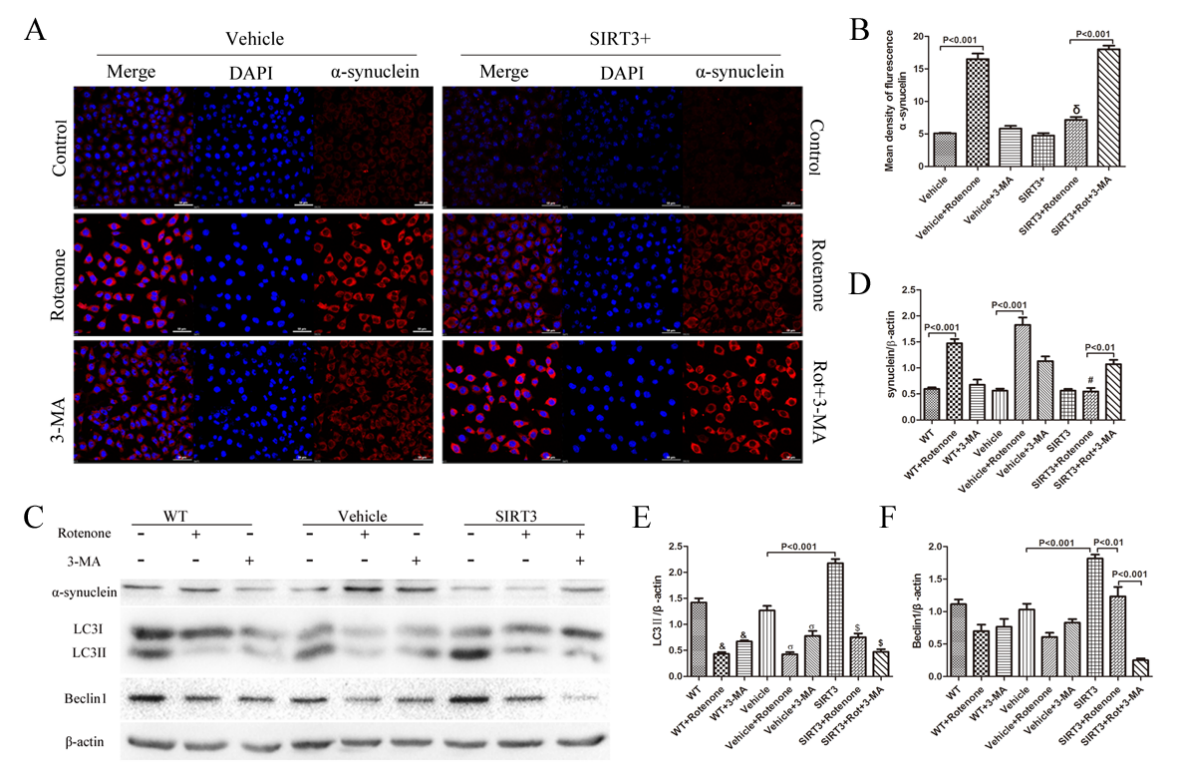
SIRT3 enhances the clearance of α-synuclein by upregulating autophagy. (A) SH-SY5Y cells with or without SIRT3 overexpression cultured in non-serum growth medium were treated with 60μM rotenone for 24h. Five millimolar 3-MA was added 1h before rotenone. The cells were immunostained for α-synuclein (red). DAPI staining was used to mark the position of the nuclei. Scale bars=50 μm. (B) A bar graph shows the quantification of mean fluorescence intensity of α-synuclein in each group. Mean ± SEM, n=3. δ: *P*<0.001 vs. Vehicle+Rotenone. (C) The cells were treated in the same way as mentioned in A, and cell lysates were prepared and analyzed by western blotting using antibodies against α-synuclein. LC3 and Beclin 1 immunoblotting were performed to test the autophagic level under rotenone or 3-MA treatment. (D, E, F) Bar graphs show the quantification of the relative level of α-synuclein, LC3II and Beclin1 in each group. β-actin is used as a loading control. Mean ± SEM, n=3. #: *P*<0.001 vs. Vehicle+Rotenone. &: *P*<0.01 vs. WT. σ: *P*<0.001 vs. Vehicle. $: *P*<0.001 vs. SIRT3.

**Figure 5.**
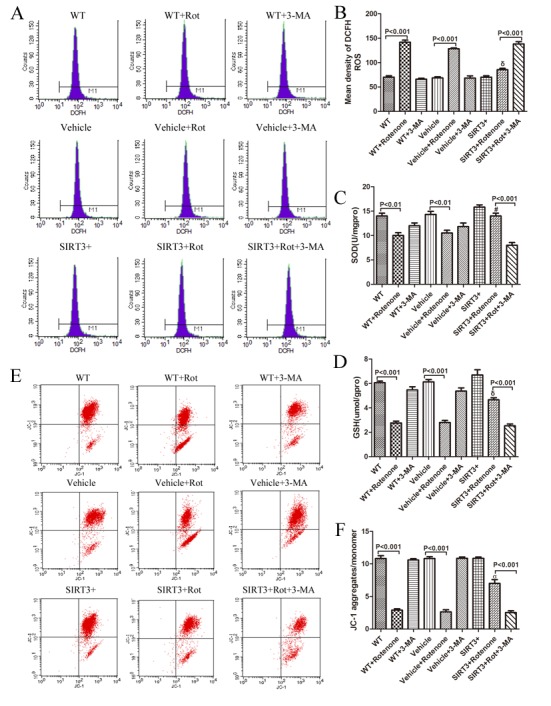
SIRT3 upregulates intracellular SOD and GSH levels, prevents rotenone-induced ROS generation and MMP collapse in SH-SY5Y cells by upregulating autophagy. The SH-SY5Y cells with or without SIRT3 overexpression cultured in serum-free growth medium were treated with 60μM rotenone for 24 h. Five millimolar 3-MA was added 1h before rotenone. The cells were harvested and stained with DCFH-DA. (A) ROS levels were measured by a fluorescence-activated cell sorter. (B) Bar graph shows the quantification of ROS levels. Mean ± SEM, n=3. δ: P<0.001 vs. Vehicle+Rotenone. The cells were collected for quantification of intracellular SOD (C), GSH (D) using the corresponding detecting kits. Mean ± SEM, n=3. #: *P*<0.05 vs. Vehicle+Rotenone. δ: *P*<0.001 vs. Vehicle+Rotenone. (E) MMP was measured by JC-1 aggregates/monomer using a flow cytometer. (F) The bar graph shows the quantification of JC-1 aggregates/monomer levels in each group. Mean ± SEM, n=3. σ: *P*<0.001 vs. Vehicle+Rotenone.

**Figure 6.**
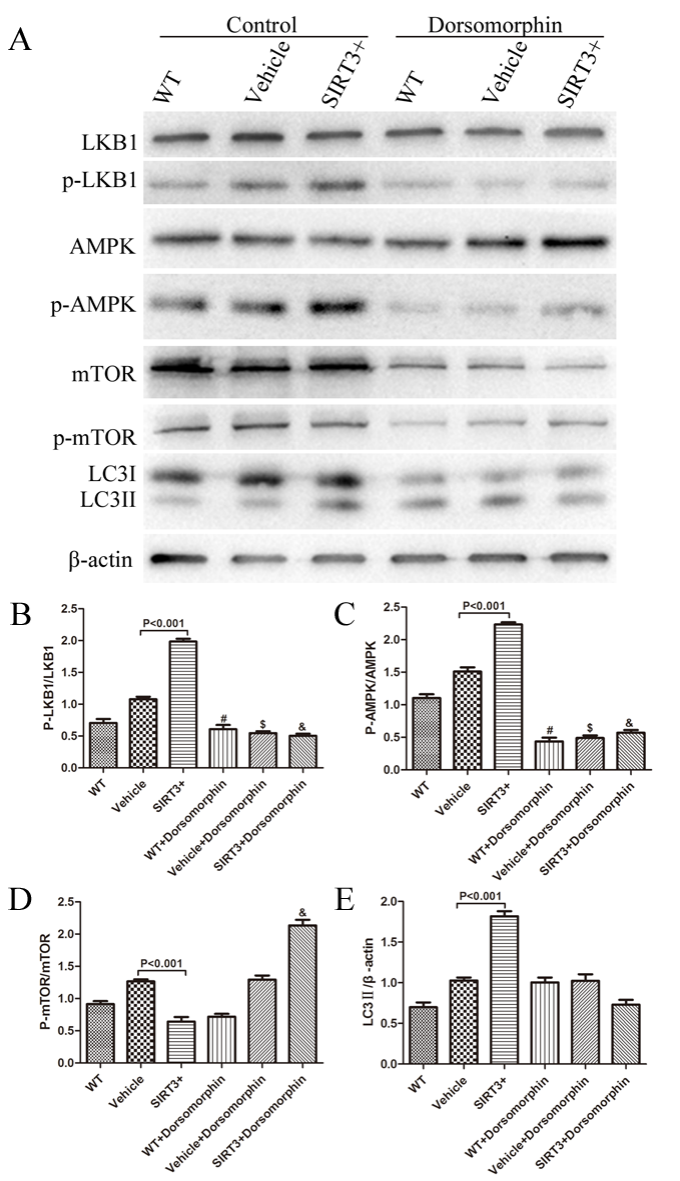
SIRT3 induces autophagy through the LKB1-AMPK-mTOR pathway. (A) The SH-SY5Y cells with or without SIRT3 overexpression were treated with 40 μM Dorsomorphin or control for 100 min. The cell lysates were analyzed by western blotting with the indicated antibodies. Data show the quantification of the p-LKB1/LKB1 ratio (B), p-AMPK/AMPK ratio (C), p-mTOR/mTOR levels (D) and LC3II (E). β-actin is used as a loading control. #: *P*<0.001 vs. WT, $: *P*<0.001 vs. Vehicle, &: *P*<0.001 vs. SIRT3+. Mean ± SEM, n=3.

### SIRT3 enhances clearance of α-synuclein by up-regulating autophagy

Given that α-synuclein accumulation is one of the pathologies of PD, we examined the relationship between

SIRT3 and α-synuclein. Immunofluorescence staining showed that after 24 h incubation with cells, rotenone notably increased α-synuclein expression in the Vehicle cells, while α-synuclein levels were compromised in SIRT3-overexpression cells after rotenone treatment. The addition of 3-MA abolished this amelioration effect of SIRT3, and the α-synuclein positive fluorescence intensity increased distinctly in the SIRT3+Rotenone+3-MA-group compared with the SIRT3+Rotenone-group (Fig. 4A, 4B). After the quantification at cellular level using fluorescence staining, we performed western blot analysis to confirm the SIRT3 and 3-MA effect on synuclein expression. α-synuclein levels almost tripled in the WT and Vehicle cells after rotenone treatment, while in the SIRT3 cells, its expression showed almost no change following rotenone treatment. Again, after addition of 3-MA, the effect of SIRT3 was blocked (Fig. 4C, 4D). Western blot analyses for LC3II and Beclin 1 were performed to test the autophagic levels after rotenone and 3-MA treatment. LC3II expression decreased significantly in the WT, Vehicle and SIRT3 groups following rotenone and 3-MA treatment. Beclin1 showed a significant decrease in SIRT3 cells after rotenone and 3-MA addition (Fig. 4C, 4E, 4F). These results thus demonstrated the involvement of autophagy in the SIRT3-mediated clearance of α-synuclein after rotenone treatment.

### SIRT3 attenuates rotenone-induced oxidative stress and mitochondrial dysfunction by upregulating autophagy

Oxidative stress has been implicated in the propagation of neuronal dysfunction in multiple neurodegenerative diseases. Given that SIRT3 reduces the cell apoptosis percentage and increases cell viability after rotenone treatment, we next tested its effect on oxidative stress, with ROS expression indicating the oxidants level and SOD and GSH production representing the antioxidants level. After inhibition of autophagy using 3-MA (5 mM, 1h), the protective effect of SIRT3 against oxidative stress diminished when comparing the SIRT3+Rotenone+3-MA-group with the SIRT3+Rotenoe-group: ROS generation was enhanced (Fig. 5A, 5B) and intracellular SOD (Fig. 5C) and GSH levels (Fig. 5D) decreased. MMP was used to evaluate the degree of mitochondrial dysfunction. Flow cytometry results showed that there was a significant MMP reduction in the SIRT3+Rotenone+3-MA-group compared with the SIRT3+Rotenone-group. These results again demonstrated the involvement of autophagy in the neuroprotective effects of SIRT3, which attenuates rotenone-induced oxidative stress and mitochondrial dysfunction.

### SIRT3 upregulates autophagy through the LKB1-AMPK-mTOR pathway

AMPK is a positive regulator of autophagy via mTOR or ULK1[28]. We therefore focused our attention on the involvement of the AMPK signaling pathway in SIRT3-mediated autophagy activation in SH-SY5Y cells. The results showed that AMPK activation (phosphorylation) was notably increased in SIRT3+ cells (Fig. 6A, 6C). The activity of AMPK is known to be regulated by an upstream kinase, LKB1. We therefore examined LKB1 phosphorylation, and found that, similar to AMPK, LKB1 phosphorylation was also significantly enhanced in SIRT3+ cells (Fig. 6A, 6B), and thus suggesting that SIRT3 had the capacity to upregulate the activity of LKB1 and AMPK. Additionally, mTOR phosphorylation was reduced (Fig. 6A, 6D) and LC3II was remarkably increased (Fig. 6A, 6E) in SIRT3+ cells compared with Vehicle cells. Thus, we extended our hypothesis that SIRT3 may induce autophagic level acceleration through the LKB1-AMPK-mTOR signaling pathway.

Then, we used an AMPK inhibitor to continue to clarify the relationship between the LKB1-AMPK-mTOR signaling pathway and autophagy induction. Dorsomorphin, an AMPK inhibitor, can act as a reversible and ATP-competitive inhibitor of AMPK [29] and was used here. After delivery of Dorsomorphin (40 μM, 100 min), AMPK phosphorylation reduced in WT, Vehicle and SIRT3 cells (Fig. 6A, 6C). At the same time, mTOR phosphorylation increased significantly in the SIRT3+ group (Fig. 6A, 6D). We also observed a significant decrease in LC3II expression after Dorsomorphin treatment in SIRT3+ cells (Fig. 6A, 6E).

## DISCUSSION

**Figure 7.**
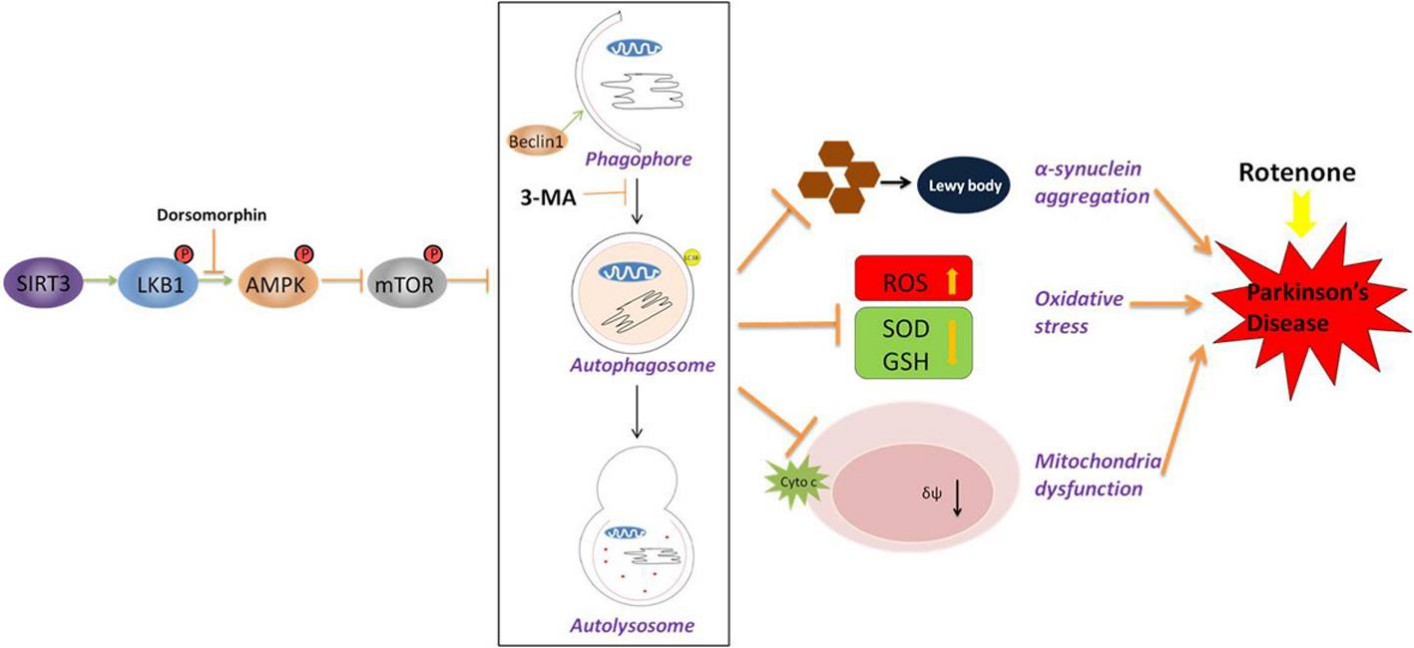
A schematic model for SIRT3-induced autophagy and its underlying mechanism in SH-SY5Y cells following rotenone treatment. SIRT3 overexpression leads to an acceleration of LKB1 activation (phosphorylation) and downstream AMPK activation, thus decreasing mTOR phosphorylation and initiating an autophagic response. In a rotenone-induced Parkinson cell model, there is enhanced α-synuclein aggregation, accelerated oxidative stress and increased mitochondria dysfunction. With SIRT3 overexpression and induced autophagic levels, these injuries are ameliorated. Blocking autophagy using 3-MA abolished the protective effect. Our results suggest that SIRT3 protects cells from rotenone by generating autophagy. The LKB1-AMPK-mTOR pathway is involved in the generation.

In this study, we report that SIRT3 protects rotenone-induced SH-SY5Y cell damage by upregulating autophagy, where it increases cell viability and decreases cell apoptosis, attenuates the accumulation of α-synuclein, increases intracellular SOD and GSH levels, ameliorates rotenone-induced ROS generation, and preserves MMP reduction in SH-SY5Y cells. These effects could be blocked upon the inhibition of autophagy. We also demonstrated that the modulation of autophagy by SIRT3 is through the activation of the LKB1-AMPK-mTOR signaling pathway (Fig. 7).

An important finding reported here is the ability of SIRT3 to induce autophagy activity. Autophagy, which is the degradation of cytosolic components in lysosomes, has been recognized for more than five decades. A large number of studies have indicated that autophagy plays an important neuroprotective role in neurodegenerative diseases, including PD [4, 30, 31]. The role of SIRT3 in autophagy induction in SH-SY5Y cells has not been assessed before: however, previous studies have shown that autophagy can be induced in SH-SY5Y cells by other members of the sirtuin family, SIRT1 and SIRT2 [32-34]. We found that SIRT3 induces Beclin 1 expression and increases LC3II levels. Bafilomycin A1, an autophagosome-lysosome fusion inhibitor, was used to confirm this result. In addition, after SIRT3 silencing, autophagy levels decreased significantly. Together with the electron microscopy images, these data strongly suggest that SIRT3 could upregulate autophagy levels in SH-SY5Y cells. Another important finding reported here is the involvement of autophagy in the neuroprotective effects of SIRT3 through the MTT assay and apoptosis analysis. It should be noted that although in PD, autophagy plays beneficial effects on the disease’s progression, autophagy can exert both beneficial and aggravating effects on different diseases. Autophagy seemed to have a dual role, which not only could be cytoprotective but also could lead to cell death. This paradoxical role of autophagy always depends on the time and the extent of the induction of autophagy[35].

Widespread accumulation of the α-synuclein protein in the form of Lewy bodies and Lewy neuritis is a neuropathological hallmark of PD [1]. The autophagy pathway was reported to be relevant to α-synuclein clearance [8]. Autophagy-lysosome pathway (ALP) inhibition significantly potentiates the toxicity of aggregated α-synuclein species in mice and cell models[36]. The conditional knock-out of the autophagy-related gene Atg7 leads to the accumulation of α-synuclein [37]. The cytoplasmic acetylation of α-synuclein is implicated in the neuroprotection against PD [38]. Among these mitochondrial deacetylase sirtuins, SIRT3 plays an important role[39]. In our study, we found that SIRT3 diminished α-synuclein aggregation via the upregulation of autophagy. In accord with the previous report that 3-MA can suppress proteolysis independently of autophagy [40], we also observed an increase trend of synuclein expression following 3-MA treatment both in WT and Vehicle cells.

Cumulative oxidative stress has been known to be a key factor in the acceleration of the aging process and the development of neurodegenerative disorders [41]. Defects in mitochondrial dynamics, and the generation and presence of ROS in many cases are associated with neurodegenerative diseases, such as PD[42]. Mitophagy, known as a selective form of autophagy, specifically targets damaged or superfluous mitochondria for autophagic degradation and has been repeatedly shown to take part in the maintenance of a healthy mitochondrial population. Evidence has shown that SIRT3 could promote oxidative stress resistance by deacetylating lysine residues on SOD2 [43]. SIRT3 is also an essential player in enhancing the mitochondrial glutathione antioxidant defense system during caloric restriction (CR)[44]. We found that SIRT3 blocked rotenone-induced ROS generation and enhanced the antioxidant system through autophagy modulation. The MMP collapse was ameliorated by SIRT3 through autopahgy mediation. These results suggested that the protective effect of SIRT3 on mitochondria was via the activation of autophagy.

How does SIRT3 improve autophagy levels? Studies were carried out to understand the mechanism of the ability of SIRT3 to activate autophagic flux. The SIRT1/SIRT3-FOXO3a pathway is activated by Liraglutide to promote autophagy in non-alcohol fatty liver disease [22]. SIRT3 could activate the LKB1-AMPK pathway in a cardiac hypertrophic mouse model[23]. AMPK acts on a downstream target, the mammalian target of rapamycin (mTOR) kinase, to positively regulate autophagy[45]. Several upstream kinases, including liver kinase B1 (LKB1, which is activated by energy depletion), can activate AMPK by phosphorylation [46]. In this study, we found that in the SH-SY5Y cell line, SIRT3 overexpression could induce the LKB1-AMPK-mTOR pathway, which may be part of the mechanism by which SIRT3 promotes autophagy. However, more evidence regarding SIRT3 and these kinases in a rotenone-induced injury model is needed. In addition, we also observed a decrease in LKB1 phosphorylation after Dorsomorphin treatment. The interaction of AMPK and LKB1 may be one possible explanation. Another reason for this may be the nonspecific effect of Dorsomorphin, which could also inhibit a number of other protein kinases [47]. Two of these protein kinases, Lck [48] and Yes [49], have been reported to be functionally important for LKB1 phosphorylation. Lastly, we cannot rule out that there is other mechanism for the neuronal protective effect of SIRT3 on rotenone-induced damage.

We previously reported the protective effect of SIRT3 on a Parkinson’s Disease cell model [17]. This paper focuses on the mechanism of the protective effect. Innovations of our study are: (1) although several in vivo and in vitro reports have reported the positive regulation of SIRT3 on autophagy [21, 50, 51], it is not clear whether there is link between the protective effect of SIRT3 on PD. We found in this study that SIRT3 exerts a protective effect on a rotenone-induced PD cell model by enhancing autophagy; (2) Behrends et al. suggested that AMPK might act as an activator to promote autophagy via mTOR or through an interaction with ULK1[24]. However, studies supporting this theory remain to be conducted. Our study demonstrated for the first time that SIRT3 enhances autophagy through the LKB1-AMPK-mTOR pathway.

Our study provides new data for a better understanding of the protective role played by SIRT3 in a rotenone-induced PD cell model. To the best of our knowledge, this is the first report describing a neuroprotective role of SIRT3 on a PD cell model through autophagy induction and its related mechanism. However, some limitations to this study should be noted: (1) we used an in vitro model of PD induced by rotenone in human dopaminergic SH-SY5Y cells. Although rotenone as an inhibitor of mitochondrial complex I, can induce neuronal dysfunction and neurodegeneration, which mimics the pathology of PD, there may be different micro-environments and various other factors that could limit the reliability of in vitro study when compared with in vivo study or clinical trials. It is our goal to perform in vivo experiments for the next step based on the in vitro discovery. (2) We used 3-MA to inhibit autophagy to verify its protective effect. Although we used LC3 and Beclin1 to identify the inhibition, the dual role of 3-MA in modulating autophagy is still a concern. Knockdown of ATG protein is a more direct way to inhibit autophagy, which is a more preferable method in future studies.

### Conclusion

In summary, this study demonstrates that SIRT3 induces autophagy to ameliorate rotenone-induced SH-SY5Y cell damage, inhibit intracellular α-synuclein accumulation, increase antioxidants of SOD and GSH, prevent ROS generation and ameliorate MMP reduction. The mechanism of this autophagy modulation may be via the LKB1-AMPK-mTOR pathway. Our findings pose that SIRT3 may be a promising drug target for PD treatment. Further research regarding the molecular mechanism of SIRT3 action and which stage of autophagy is modulated by SIRT3 would be required to understand whether autophagy serves as a bridge between SIRT3 and PD. These efforts will not only provide us with a deeper understanding of PD but also may lead to a new means for therapeutic intervention in neurodegenerative diseases.
